# Bridging the new drug access gap between China and the United States and its related policies

**DOI:** 10.3389/fphar.2023.1296737

**Published:** 2024-01-08

**Authors:** Xingyue Zhu, Yang Chen

**Affiliations:** ^1^ Department of Pharmacy Administration, School of Medicine and Health Management, Guizhou Medical University, Guiyang, Guizhou, China; ^2^ The Third People’s Hospital of Chengdu, Chengdu, Sichuan, China

**Keywords:** drug lag, launch delay, R&D, clinical trial, absolute lag, relative lag

## Abstract

**Introduction:** The access gap for novel pharmaceuticals between China and the developed countries is a major public health issue in China. It is crucial to understand the determinants of this gap to ensure timely access to new drugs and enhance patient health.

**Methods:** We included all new drugs approved by the US Food and Drug Administration (FDA) between 2012 and 2019, and collected their approval timings in China. Major factors of interest comprised orphan designation and expedited review pathways granted by the FDA, along with the proportion of Asian subjects in the pivotal trial supporting the FDA approval and whether the trial included study sites in China. The elapsed time from the FDA approval to the market authorization in China constituted the time-to-event outcome, and Cox proportional-hazards regression was used for multivariate analysis.

**Results:** A total of 327 new drugs were approved by the FDA between 2012 and 2019, among which 41.3% were found to be authorized in China as of 1 November 2023. The median lag time for the mutually approved drugs was 3.5 years. The Cox model found that orphan drugs had lower likelihood of being approved in China (HR = 0.59, 95% CI 0.39–0.89; *p* = 0.011), while the FDA’s Breakthrough-Therapy drugs (HR = 2.33, 95% CI 1.39–3.89; *p* = 0.001) and Fast-Track drugs (HR = 1.58, 95% CI 1.05–2.38; *p* = 0.028) had shorter lag times. In the pivotal trials that supported the FDA approvals, a higher proportion of Asian subjects was associated with faster drug entry into the Chinese market (HR = 1.02, 95% CI 1.01–1.03; *p* < 0.001), and the inclusion of study sites in China mainland was likewise conducive to reducing the drug lag (HR = 5.30, 95% CI 3.20–8.77; *p* < 0.001). After the trials with China-based sites supported the FDA approvals, 77.8% of the trials also supported the subsequent approvals in China.

**Discussion:** China’s involvement in global drug co-development can streamline clinical development, by reducing repeated trials solely in the Chinese population. This is primarily due to the openness of the Chinese drug agency towards overseas clinical data and is a positive sign that encourages global drug developers to include Chinese patients in their development plans as early as possible.

## 1 Introduction

Technological breakthroughs and medical innovations play a key role in improving patient health and saving lives. However, following the advent of a new drug, patients’ access to it is not equally guaranteed in all regions worldwide. The regulations with regard to drug research and development (R&D), approval process and price, as well as the pharma’s strategies for R&D and market access, will affect whether a new drug can be brought to the patients in a particular country and how long the patients wait (Danzon et al., 2005; Hirai et al., 2010; Wileman and Mishra, 2010). These two questions are respectively referred to as the absolute drug lag and the relative drug lag, which represent the two dimensions of the access gap for new drugs between countries (Wardell, 1973). Drug lag has been a major public health issue in many regions (Berndt and Cockburn, 2014; Poirier, 2015; Sun, 2019; Choi et al., 2023), since it is detrimental to patients’ health by precluding them from superior medications. China is reported to be afflicted with severe drug lag (Zhu and Liu, 2020; Li and Yang, 2021), with a median of 3.7-year access gap behind the United States or EU (Zhu and Liu, 2020). In response, the Chinese regulator has initiated several countermeasures since 2015. First, priory review and conditional approval are introduced to respectively reduce the time spent in the drug review process (Zhou et al., 2017) and grant early approval based on surrogate endpoints (Zou et al., 2023). Second, the drug regulatory system and the R&D environment have been improved. Implied licensing has been implemented for Investigational New Drug applications since 2017, granting automatic approval for clinical research in China in 60 days if National Medical Product Administration (NMPA), the Chinese drug agency, does not provide comments (China NMPA, 2019). Additionally, China has allowed phase Ⅰ multi-region trials (MRTs) since 2017 (Bajaj et al., 2019). Most importantly, domestic standalone clinical trials in China are also no longer a prerequisite when applying for new drug market authorization by NMPA: they can be replaced by MRTs enrolling Chinese patients, or be exempted for drugs treating rare diseases (Bajaj et al., 2019). After China joined The International Council for Harmonisation of Technical Requirements for Pharmaceuticals for Human Use (ICH) in 2017, the acceptance of overseas clinical data in drug approval decisions has been further improved (Liu et al., 2022). The existing literature has measured the absolute and relative drug lag in China (Zhou et al., 2019; Liu et al., 2022; Luo et al., 2023), but only focuses on oncology drugs, leaving other diseases understudied. Moreover, whether the recent reform efforts, particularly the relaxation of clinical research requirements, influence the drug lag in China likewise awaits answers. As the most time-consuming phase, the clinical development for new drugs is expected to be expedited after NMPA embraces overseas clinical data.

In this study, we assess the drug lag in China for new drugs approved by the US Food and Drug Administration (FDA) between 2012 and 2019, and investigate the effects of key pharmaceutical characteristics and clinical trial design features on the observed drug lag. Our work will contribute to understanding the implications of the recent regulation about adopting overseas clinical data, and help shape future policy options for both the Chinese regulator and the industry to facilitate early access to novel therapies.

## 2 Methods

### 2.1 Data

Based on the Drugs@FDA database (U.S. Food and Drug Administration. Drugs FDA, 2022), we collected all the new chemical entities and new biologics that were approved by the FDA between 1 January 2012 and 31 December 2019. This timeframe provided us with relatively sufficient time to observe the approvals of the study drugs in China and ensured that most drugs could fall under NMPA’s new policies. For each drug, the basic information was assembled: the dates of approval and submission, registration class [New Drug Application (NDA) or Biologic License Application (BLA)], orphan designation, reception of expedited review pathways (Priority Review, Accelerated Approval, Fast Track, and Breakthrough Therapy) and approved indications. Review times were defined as the days from the submission date to the approval date. The WHO’s Anatomic Therapeutic Classification (ATC) system was used to identify the therapeutic area of each drug (WHO Collaborating Centre for Drug Statistics and Methodology, 2022). To explore the effects of the sponsor’s R&D strategies, the pivotal trial(s) of each drug was identified using the disclosed review report in Drugs@FDA; and the factors of interest were the trial locations, and the proportion of Asian subjects to the total enrollment, which were obtained through ClinicalTrial.gov (ClinicalTrials, 2023) and the FDA review reports. A dummy variable for trial type was thus created according to the locations of a pivotal trial: 0 indicated no inclusion of China sites, 1 indicated inclusion of sites in China mainland, and 2 indicated inclusion of Hongkong or/and Taiwan region, China. For the proportion of Asian subjects, the number of all Asian participants was used if the Asian race was not further classified; otherwise, the total number of participants with Eastern Asian heritage, Chinese heritage, or Japanese heritage was used. When a drug approval was supported by more than one pivotal trial, the best value for trial location type variable (1 > 2 > 0) and the maximum proportion of Asian subjects were assigned to the drug. Note that the best values for the proportion of Asian subjects and the trial location did not necessarily come from the same trial.

Next, we determined whether the study drugs were approved in China, using the database of NMPA (China NMPA, 2023). For each drug approved by NMPA, the Listed Drug Database of Center of Drug Evaluation (CDE) was used to find its review report (NMPA Center of Drug Evaluation, 2022), based on which the information of pivotal trial(s) was collected. The NMPA’s pivotal trials were categorized into three types: the first was new overseas trials, referred to that all the pivotal trials were exclusively conducted outside of China and differed from those that supported the specific FDA approvals; the second was new trials with sites in China, as long as there was one such study among all the pivotal trials; and the third was the trials identical to those supporting the specific FDA approvals, provided that there was at least one such study among all the pivotal trials and the rest contained no sites in China. The absolute lag was measured by the number of drugs introduced in China and their proportion, while the relative lag was defined as the gap time between the two approval timings of the FDA and NMPA. The approval status in China for the study drugs was followed up to 1 November 2023.

### 2.2 Statistical analysis

The gap time from the FDA approval to the NMPA approval formed the time-to-event outcome. Medians with interquartile range were employed to describe the relative lag. Fisher exact test was used to compare the NMPA approval rates for drugs with different types of the FDA’s pivotal trial. The Cox proportional-hazards model was used as the multivariate analysis to examine the factors of the occurrence of drug launch in China. The main factors of interest were the proportion of Asian subjects and the location type of the pivotal trials that supported the FDA approval. We hypothesized that if a well-designed MRT contained study sites in China or included sufficient Asian participants, it would be more likely to be the common basis for both the regulatory approvals in the US and in China, according to which the drug lag would be reduced noticeably. The Cox model likewise took into account the FDA’s orphan designation and expedited review pathways. Orphan designation suggested the situation of rare conditions that were more plagued with drug accessibility issues (Yan et al., 2019). As claimed by the aim and scope of the established pathways (U.S. Food and Drug Administration, 2018a; U.S. Food and Drug Administration, 2018c; U.S. Food and Drug Administration, 2018b), we considered Breakthrough Therapy designation as a proxy for substantial clinical improvement, Fast Track designation as a proxy for serious conditions, and Accelerated Approval designation as a proxy for unverified evidence of efficacy. In the multivariate analysis, therapeutic areas were rearranged into two categories (cancer and non-cancer) to reduce potential overspecification. The approval year, as a predictor for time trend, the registration class, and the FDA review times were also included as covariates. The significance level was set to be 0.05 for 2-tailed tests, and robust standard errors were reported. Stata version 15 (StataCorp LP) was used to perform the analysis.

## 3 Results

### 3.1 New regulation in China

**FIGURE 1:**
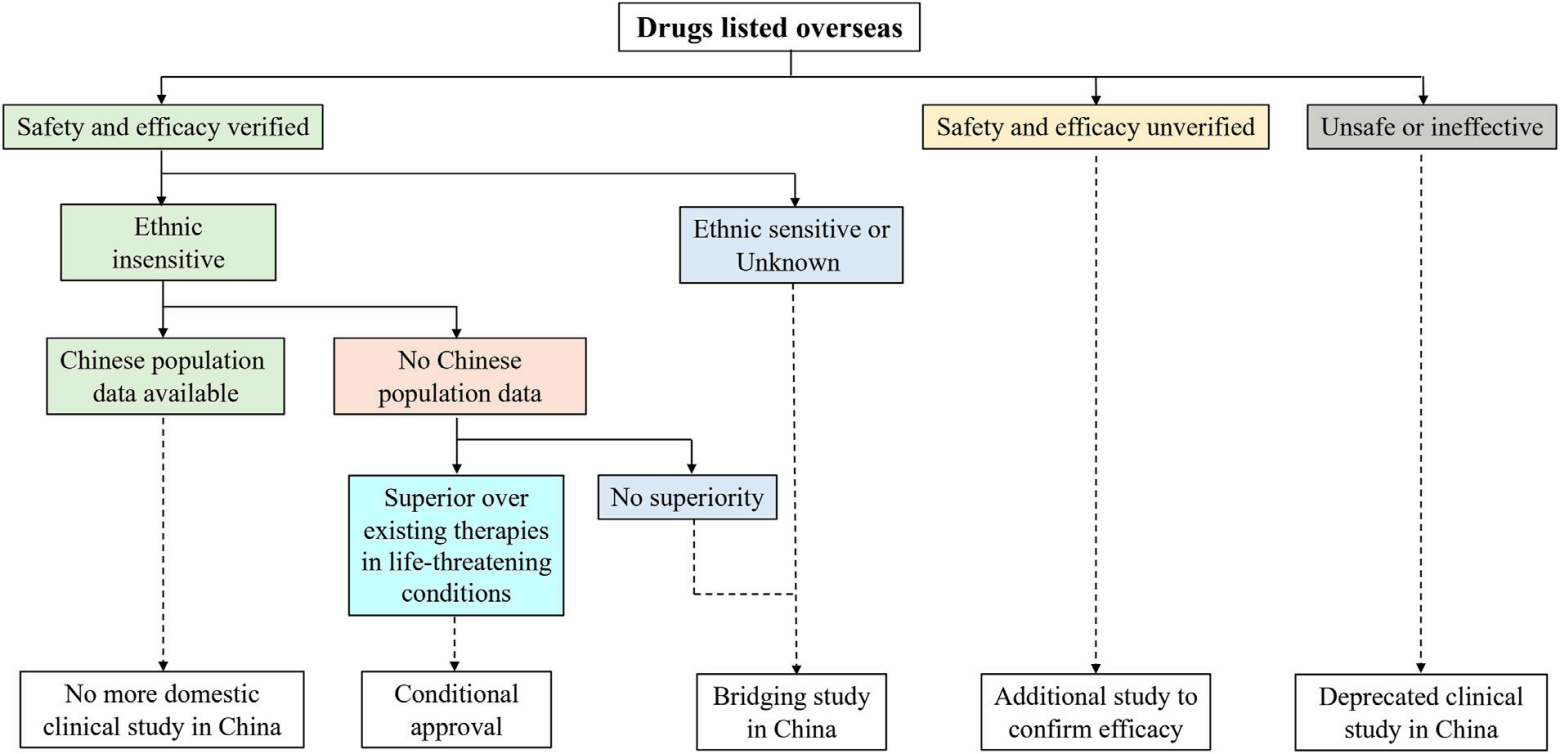
Clinical research required to obtain marketing authorization in China for drugs that have been listed overseas. To expedite access to foreign new drugs with substantial clinical interests, National Medical Products Administration (NMPA) issued *Clinical Research Technique Requirements for Drugs Listed Overseas but Not Listed in China*. Imported drugs with no predicted ethnic difference can apply for a waiver of repeated studies in Chinese patients.

To deal with the drug lag issue, NMPA has stipulated the *Clinical Research Technique Requirements for Drugs Listed Overseas but Not Listed in China* in 2020 (China NMPA, 2020), which reveals the key considerations in clinical development and approval decision for drugs that are seeking NMPA’s marketing authorization using overseas data (Figure 1). The acceptance of overseas data relies on the size of the clinical needs in China, drug safety and efficacy profiles from current global studies, and ethnic sensitivity results. If the risk-benefit balance for a specific new drug has been verified to be acceptable in the general population, and no ethnic sensitivity is identified in the Chinese population, additional domestic clinical studies in China can be expected to be reduced or waived. In the case where ethnic sensitivity raises concerns or remains uninvestigated, a bridging study in China will be required. The ethnic sensitivity analysis should be compliant with ICH E5 and ICH E17 guidelines.

### 3.2 Drug lag in China

A total of 327 new drugs were approved by the FDA between 2012 and 2019, among which 135 (41.3%) were authorized in China as of 1 November 2023 (Table 1). The absolute lags for NDAs and BLAs were close (40.8% vs. 42.7%). Only 38.0% of the orphan drugs were approved by NMPA. Drugs with Breakthrough Therapy and with Accelerated Approval had higher proportions of NMPA approvals than those with the other two expedited pathways. With regard to the ATC categories, the cardiovascular system had the least absolute lag (53.8%) while the antiparasitic products, insecticides and repellents had no approval in China. The median review times of the FDA was 301 days (IQR, 238–365).

**TABLE 1:** Summary of new drugs approved by the FDA in 2012–2019.

Variables	All drugs, N (%)	Drugs approved in China, N (%)
Total	327 (100)	135 (41.3)
Drug-level		
Registration class		
NDA	245 (74.9)	100 (40.8)
BLA	82 (25.1)	35 (42.7)
Orphan drug	142 (43.4)	54 (38.0)
Expedited pathway		
Priority review	190 (58.1)	78 (41.0)
Fast track	122 (37.4)	51 (41.8)
Accelerated approval	44 (13.5)	22 (50.0)
Breakthrough therapy	70 (21.4)	35 (50.0)
ATC		
A Alimentary tract and metabolism	39 (11.9)	16 (41.0)
B Blood and blood forming organs	16 (4.9)	7 (43.8)
C Cardiovascular system	13 (4.0)	7 (53.8)
D Dermatologicals	11 (3.4)	2 (18.2)
G Genitourinary system and sex hormones	7 (2.1)	2 (28.6)
H Systemic hormonal preparations, excluding sex hormones. and insulins	6 (1.8)	1 (16.7)
J Anti-infectives for systemic use	44 (13.5)	22 (50.0)
L Antineoplastic and immunomodulating agents	108 (33.0)	60 (55.6)
M Musculoskeletal system	7 (2.1)	2 (28.6)
N Nervous system	34 (10.4)	6 (17.6)
P Antiparasitic products, insecticides and repellents	5 (1.5)	0
R Respiratory system	12 (3.7)	4 (33.3)
S Sensory organs	8 (2.4)	2 (25.0)
V Various	17 (5.2)	4 (23.5)
FDA review times, median (IQR), days	301 (238–365)	245 (202–365)
NMPA review times, median (IQR), days	NA	391 (296–511)
Launching lag time, median (IQR), days	NA	1,274 (821–1757)
Trial-level		
Proportion of Asian subjects, median (IQR),%	4.6 (1.3–17.1)	13.6 (3.8–27.2)
Trial type		
No China sites	233 (71.2)	76 (32.6)
With sites in China mainland	31 (9.5)	28 (90.3)
With sites in Hongkong/Taiwan, China	63 (19.3)	31 (49.2)

Abbreviations: NDA, new drug application; BLA, biologic license application; ATC, anatomic therapeutic classification system; IQR, inter-quartile range; FDA, the US, food and drug administration; NMPA, china national medical product administration; NA, not applicable.

Features of the pivotal trials that supported the FDA approvals were summarized in Table 1, as well. The median proportion of Asian subjects in the pivotal trials for all drugs was 4.6% (IQR, 1.3%–17.1%). However, the drugs approved in China enrolled more Asian subjects, with a median proportion of 13.6% (IQR, 3.8%–27.2%). Most of the drugs were supported by trials without enrolling China sites [233 (71.2%)], among which only 32.6% were approved by NMPA; whereas drugs with study sites in China mainland had a much larger proportion of NMPA approvals (90.3%).

**FIGURE 2:**
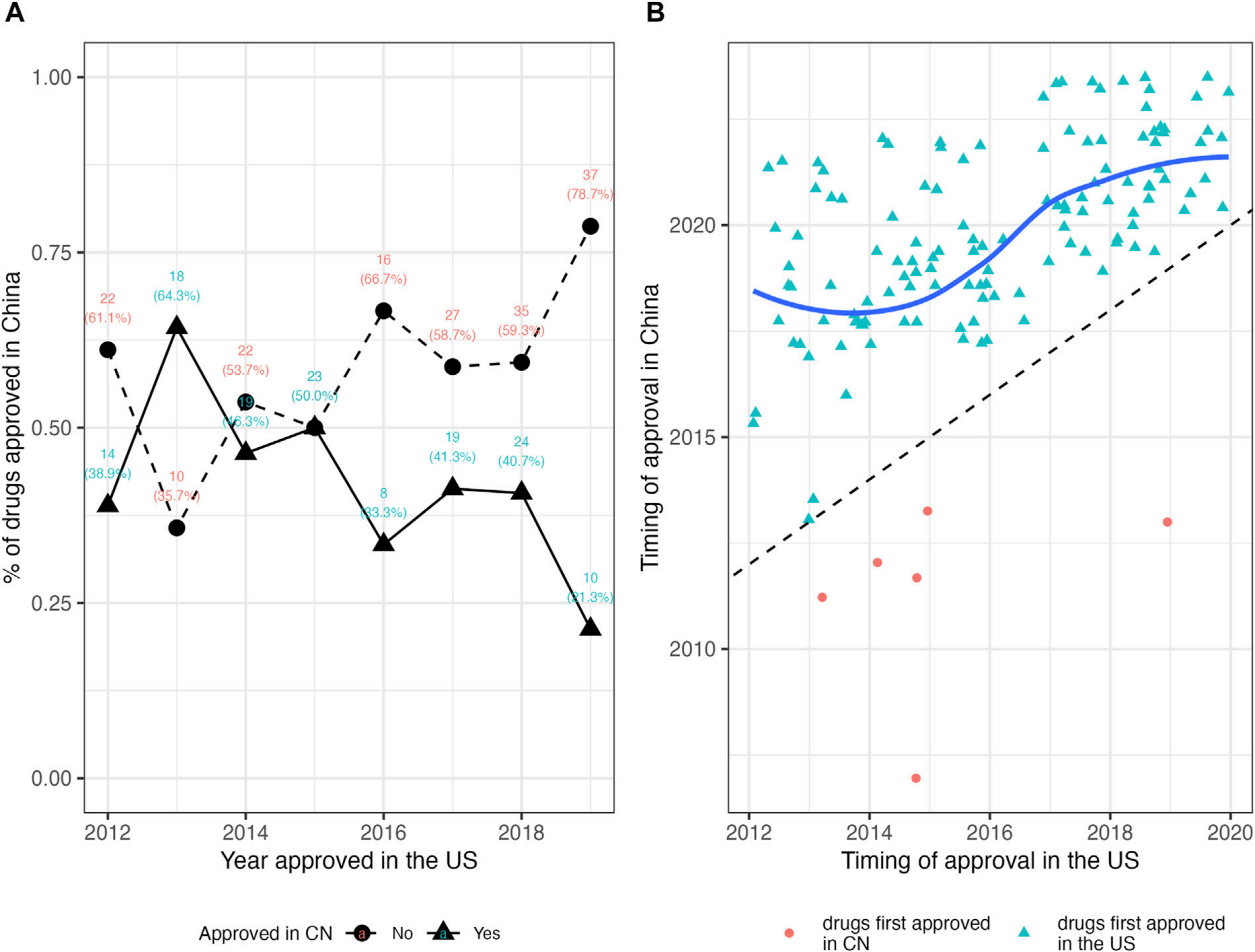
The drug lag in China for new drugs approved by the FDA in 2012–2019. **(A)** The absolute lag. Triangles denote the number and proportion of drugs approved in China, while circles denote the number and proportion of drugs not approved in China. A lower proportion of drugs approved in China indicates the severer absolute lag. **(B)** The relative lag. Red circles denote the drugs first approved in China, while light blue triangles denote the drugs first approved in the United States. The fitted line in dark blue is constructed by the LOWESS (locally weighted scatterplot smoothing) method, which indicates the tendency of the relative drug lag. The oblique dash line indicates the case of no lag (the FDA approval and the NMPA approval are simultaneous), and symbols and the fitted line that approach the oblique line present shorter lag time. CN, China; NMPA, National Medical Products Administration.

For the 135 drugs approved in China, NMPA took a slightly longer duration in drug review (median: 391 days; IQR, 296–511) as compared to the FDA. The median lag time was 1,274 days (3.5 years) (IQR, 821–1757). Among the 135 mutually approved drugs, 6 were approved in China prior to the United States, with the lag time ranging from −2,856 days (−7.8 years) to −623 days (−1.7 years); while the remaining 129 drugs were first approved in the United States, leaving a median lag of 1,320 days (3.6 years) in China (IQR, 916–1789) with a range from 25 days to 3,301 days. Figure 2 plotted the time trend of the drug lag. The absolute lag was more severe for drugs newly approved by the FDA (Figure 2A). The relative lag presented a tendency of amelioration over time, as shown by the reduced gap between the fitted line and the oblique line in Figure 2B. However, the gap remained visible, and continuing implementation of current or new policies would be needed.

### 3.3 Factors in drug lag

The results of multivariate analysis were summarized in Table 2. The registration class and therapeutic area had no detectable effect on the drug lag. However, orphan drugs had a reduced likelihood of being introduced in China (HR = 0.59, 95% CI 0.39–0.89; *p* = 0.011) as compared to non-orphan drugs. Breakthrough Therapy (HR = 2.33, 95% CI 1.39–3.89; *p* = 0.001) and Fast Track (HR = 1.58, 95% CI 1.05–2.38; *p* = 0.028) were associated with higher chances of being licensed by NMPA. Accelerated Approval did not seem to significantly correlate to the drug approval in China, nor did Priority Review. The trend of the access gap was observed to improve over time (HR = 1.17, 95% CI 1.05–1.29; *p* = 0.003). The key features of the pivotal trials supporting the FDA’s approvals also exerted some influence on the new drug’s launch in China. One more percent in the proportion of Asian subjects in the trial was associated with a 2% increase in the chance of being marketed in China (HR = 1.02, 95% CI 1.01–1.03; *p* < 0.001). Including study sites in China mainland in the trial was another strong driver for faster drug access to Chinese patients (HR = 5.30, 95% CI 3.20–8.77; *p* < 0.001), but trials with sites in Hongkong/Taiwan region did not present such an effect.

**TABLE 2:** Factors to the drug lag in China for new drugs approved by the FDA in 2012–2019.

Variable	HR	Robust SE	*p* value	95%CI
Registration class				
NDA	1 [Reference]			
BLA	0.92	0.19	0.675	0.61–1.37
Indication				
Non-cancer	1 [Reference]			
Cancer	1.21	0.30	0.437	0.75–1.96
Orphan drug				
0	1 [Reference]			
1	0.59	0.12	**0.011**	0.39–0.89
Accelerated Approval				
0	1 [Reference]			
1	1.09	0.36	0.788	0.57–2.07
Priority Review				
0	1 [Reference]			
1	0.85	0.23	0.542	0.49–1.45
Fast Track				
0	1 [Reference]			
1	1.58	0.33	**0.028**	1.05–2.38
Breakthrough Therapy				
0	1 [Reference]			
1	2.33	0.61	**0.001**	1.39–3.89
Year approved in the United States	1.17	0.06	**0.003**	1.05–1.29
FDA review times	1.00	<0.01	0.767	1.00
Asian subject Proportion in pivotal trial	1.02	<0.01	**<0.001**	1.01–1.03
Trial type				
No China sites	1 [Reference]			
With sites in China mainland	5.30	1.36	**<0.001**	3.20–8.77
With sites in Hongkong/Taiwan, China	1.19	0.30	0.488	0.73–1.95

Abbreviations: HR, hazard ratio; SE, standard error; CI, confidence interval; NDA, new drug application; BLA, biologic license application. The bold values indicate statistical significance.

**FIGURE 3:**
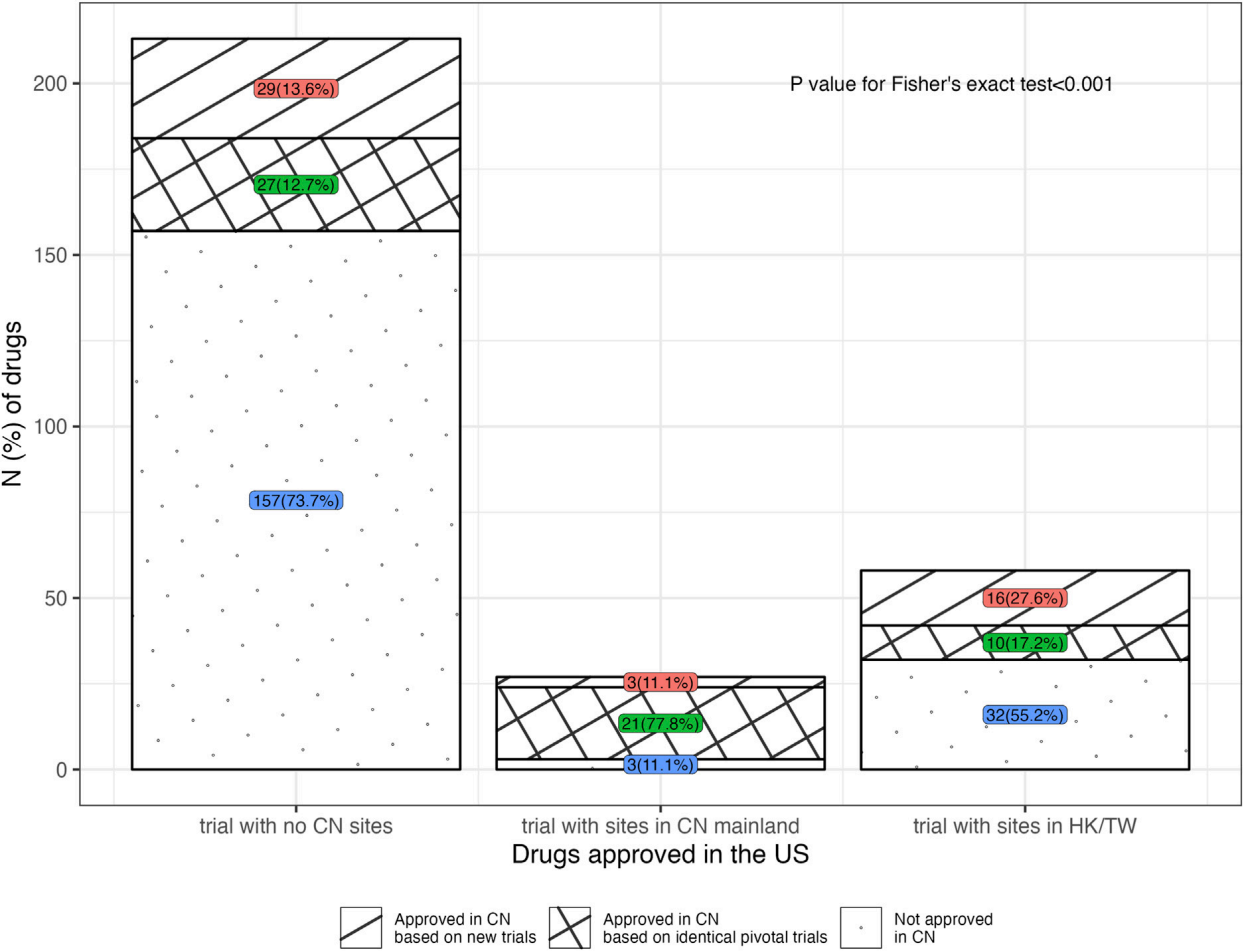
Distribution of drugs approved in the United States, in terms of the location type of the FDA’s pivotal trial. Note, 20, 4 and 5 drugs were respectively excluded due to a lack of CDE review reports in the groups for trial with no China sites, trial with sites in China mainland, and trial with sites in Hongkong/Taiwan, China. CN, China; HK, Hongkong; TW, Taiwan.

**FIGURE 4:**
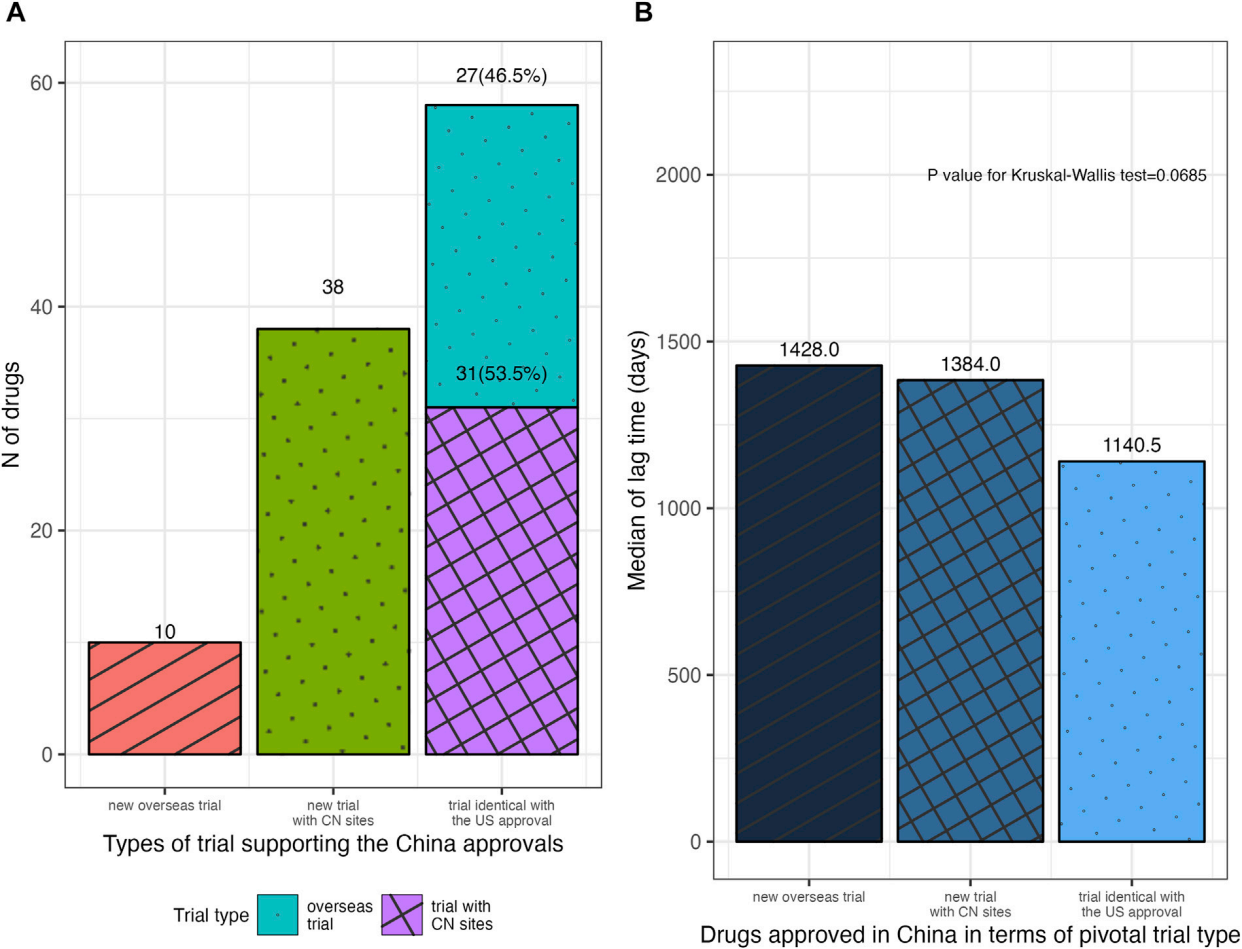
Distribution of drugs approved in China in terms of the type of NMPA’s pivotal trial. **(A)** Drugs approved in China with different types of NMPA’s pivotal trial. **(B)** The median lag time for drugs approved in China, according to the type of NMPA’s pivotal trial. NMPA, National Medical Products Administration; CN, China.

Among the 135 drugs approved in China, 106 ones had open review reports in the CDE database, which informed the analysis of the types of NMPA’s pivotal trials. Figure 3 illustrated the NMPA approval rates for drugs with different types of the FDA’s pivotal trial. According to the type of trial supporting the FDA approvals, 77.8% (21 in 27) of the drugs with sites in China mainland in the FDA’s pivotal trials were approved by NMPA on the identical trials, requiring no more Chinese data; while in the case where drugs were with no Chinese data or with only Hongkong/Taiwan data, the identical trials merely supported 12.7% (27 in 213) and 17.2% (10 in 58) of these drugs to be approved by NMPA (*p* < 0.001). Further analysis of the NMPA’s pivotal trials was in Figure 4. Of the 106 drugs, 38 (35.8%) were approved based on new trials with sites in China, 58 (54.7%) were approved based on identical trials that supported the FDA approvals, and only 10 (9.4%) were on the basis of new overseas trials. Of the 58 ones supported by identical trials, more than half [31 (53.5%)] had trials with sites in China (including Hongkong/Taiwan) as the clinical basis for approval, while the rest [27 (46.5%)] were approved solely on overseas clinical data (Figure 4A). In terms of the length of lag time, drugs approved based on identical trials had the shortest launch delay (Figure 4B). In short, NMPA has recently shown openness towards foreign data and has frequently adopted MRTs for imported drugs.

## 4 Discussion

Our work assessed the access gap for new drugs in China. Less than half of the drugs approved by the FDA during 2012–2019 were licensed in China, with a median lag time of 3.5 years and a maximum of 9.0 years. The measured relative drug lag was found to be larger than that reported in the recent research on oncology drugs in China (2.7-year lag behind the United States in 2016–2021) (Luo et al., 2023), which may suggest the continuous improvement of the access gap over time. However, it is still worth noting the constant gap in accessibility for innovative medications between patients in China and developed countries. Delayed access to cutting-edge technologies will compromise patient health, and raise the opportunity cost of inferior therapies, which is the loss of health benefits derived from these new technologies. Notwithstanding that the pharmaceutical industry in China is growing rapidly (McCall, 2021; Zhong et al., 2022), me-too innovations dominate the domestic companies (Li et al., 2022; Zhang et al., 2023). First-in-class new drugs still resort to imported products.

Clinical development lag can remarkably contribute to the drug access gap (Ni et al., 2017). In response, China has emphasized reforms in its regulatory and clinical development environment, the achievements of which are preliminarily demonstrated by this work. Accepting global studies as the basis for marketing approval has allowed for earlier entry of imported drugs: drugs with China-based sites in the FDA’s pivotal trials were associated with a five-fold likelihood to obtain the approval by NMPA, among which 77.8% were indeed licensed in China on the basis of these very trials. NMPA is increasingly adopting MRTs in its approval decision-making. With the open mind of the regulator, it can be anticipated that more ground-breaking technologies will reach Chinese patients at a higher speed. Moreover, it was found that more Asian subjects in the FDA’s trials were also helpful in reducing the drug lag. Some global studies did not include Chinese patients, but Asian participants from other regions are still valuable to clarify ethnic sensitivity concerns. Upon that ethnic sensitivity is demonstrated to be of little concern, new domestic studies in China can be streamlined or exempted, which is able to lead to faster approvals for these drugs. The findings are positive signals for global developers to build Chinese cohorts into their global development plans as early as possible.

The favorable results for Breakthrough-Therapy drugs and Fast-Track drugs proved the efforts of NMPA to meet its commitments to fulfill the therapeutic gap. Although the Breakthrough Therapy and Fast Track designations were granted by the FDA, they could serve as the proxy for the drug’s potential substantial clinical benefits in seriously debilitating diseases with unmet clinical needs. The expedited access of Breakthrough-Therapy drugs and Fast-Track drugs to the Chinese market might be attributed to the more flexible regulatory requirements and a faster review process for the drugs with clinical salience. E.g., despite ethnic variations, drugs addressing urgent medical needs can still gain approval in China, on the condition that post-marketing studies should be conducted (China NMPA, 2020). Accelerated Approval often involves indefinite evidence at approval, which might pose challenges to the drug agency in its assessment of the true risk-benefit balance (Breckenridge and Liberti, 2018). However, with a lengthy lag time, the evidence would be continuously strengthened, and the negative effect of uncertainty could diminish.

The promising solutions to the drug lag lie in the global drug co-development and synchronized applications. Nowadays, NMPA has issued the guideline for foreign sponsors to effectively use their existing global data and to save the cost of additional efficacy confirmation in the Chinese populations exclusively. To tackle the root of new drug access gap, a guideline for China’s involvement in global co-development will be helpful, through which the agency’s major concerns can be delivered to the industry: the key features of trial design (e.g., sample size of Chinese participants), the feasibility of pooled regions or pooled subpopulations, the estimation of regional treatment effects and so on. Notably, with the increasing use of surrogate endpoints in drug development, how the regulator defines the “reasonability” of a surrogate is important to the industry and the patients who receive the therapies with uncertain benefits. Surrogate endpoints are supposed to “reasonably predict clinical benefits” (U.S. Food and Drug Administration, 2018a) and hence enable early drug approval to save the time required to confirm the benefits. However, many surrogates have been found to be not valid or of unknown validity, as their correlations with true clinical benefits (e.g., the overall survival) are weak or unclear (Gyawali et al., 2020; Walia et al., 2022). Drugs approved on such weak or unvalidated surrogates are likely to fail in substantiating the improvement in overall survival, leading to risks outweighing benefits. As such, the criteria of a surrogate endpoint to be valid as the base for regulatory approval are critical to strike the tradeoff between fast market entry and safe drug access, and are also helpful in informing developers’ R&D strategies. It is also valuable of the coordination mechanism among the major global drug regulators to provide consistent and transparent guidance for MRT. In addition, NMPA’s concurrent drug review program with its international counterparts can be taken into account. Project Orbis is an international program allowing for simultaneous drug submission, review, and decision across agencies in multiple countries, including the US FDA, the Australian agency, and the Canadian agency (U.S. Food and Drug Administration, 2023). Such a synchronized drug review system, analogous to Project Orbis, can be a future policy alternative in the East Asian region (China mainland, Hongkong, Taiwan, and Japan) to coordinate the new drug approval process across the districts where populations share similar pharmacogenetical characteristics (Bajaj et al., 2019; Zhou et al., 2019).

The drug lag is affected not only by the regulations but also by the company’s strategy and investment plan. Orphan drugs were found to suffer more from the access gap issue. One important factor is that lack of knowledge, misdiagnosis or missed diagnosis, and inadequate treatment are prevalent for rare conditions in China (Li et al., 2021a; Ying et al., 2021), and hence the market for these conditions is unclear. This can undermine the market attractiveness for foreign companies. Besides, orphan designation and its matched incentives are still absent in the Chinese regulatory system. The Chinese government has issued the lists of Urgently Needed Overseas Drugs to call for drug applications (Li et al., 2021b). Drugs on the lists are eligible for expedited review pathways (Su et al., 2023). However, the present lists contain a limited number of drugs; and unlike the orphan designation of the FDA (U.S. Food and Drug Administration, 2022), the lists do not involve favorable policies for financial support and market exclusivity. To further arouse the interest of pharmas in order to improve access to orphan drugs, a comprehensive mechanism ought to be established.

Limitations of our analysis are inevitable. We only assessed the access gap in comparison to the United States. Besides, we did not include new indications of marketed drugs. Our follow-up period was limited, beyond which the launch of drugs may yet occur. Our study period covered the very time when China’s policy landscape fast evolved, and there might be some drugs subject to older regulations, which would make our results to be underestimated. Factors related to pharma companies were considered little in our study, e.g., the company scale and the utilization of out-licensing. Including China in global trials can indicate a company’s interest in the Chinese market, as well. In future research, the effects of the company’s strategies on the drug lag need to be ascertained.

## 5 Conclusion

The drug access gap between the United States and China is a persistent public health issue in China, particularly for drugs targeted in rare conditions. However, NMPA has been striving to reduce the gap and has made notable strides. The engagement of China in global co-development of new drugs and the synchronized drug applications across countries will benefit both Chinese patients and pharmaceutical innovators worldwide, but which necessitates more intensive efforts and collaboration among all relevant stakeholders.

## Data Availability

The original contributions presented in the study are included in the article/Supplementary Material, further inquiries can be directed to the corresponding author.
